# Supplementary material for: Rehabilitation status of children with cerebral palsy in Bangladesh: Findings from the Bangladesh Cerebral Palsy Register
Source: PLoS One. 2021 May 3;16(5):e0250640. doi: 10.1371/journal.pone.0250640 (PMC8092763; doi:10.1371/journal.pone.0250640)
Supplement: Attachment — Submitted filename: Rebuttal Letter_BCPR Rehab_05.04.21_V2.docx [file pone.0250640.s002.docx]

05 April 2021

Professor Joerg Heber

Editor-in-Chief

PLOS ONE

RE: Manuscript ID PONE-D-21-00848

**Title:** **Rehabilitation status of children with cerebral palsy in Bangladesh: findings from the Bangladesh Cerebral Palsy Register**

We would like to thank the academic editor and the reviewers for their constructive feedback on our manuscript titled ‘Rehabilitation status of children with cerebral palsy in Bangladesh: findings from the Bangladesh Cerebral Palsy Register’. We have incorporated all of the Academic Editor’s and Reviewers’ feedback into our revised manuscript. Additionally, all statistical methods employed in this study were re-performed and reviewed. The errors and inconsistencies identified during the review have been corrected. Please see below our point-to-point responses to the editor’s and reviewers’ comments and a detailed description of the additional changes that we have made as part of the rebuttal.

**Editor:**

**Comment-1:** Please review your reference list to ensure that it is complete and correct. If you have cited papers that have been retracted, please include the rationale for doing so in the manuscript text, or remove these references and replace them with relevant current references. Any changes to the reference list should be mentioned in the rebuttal letter that accompanies your revised manuscript. If you need to cite a retracted article, indicate the article’s retracted status in the References list and also include a citation and full reference for the retraction notice.

**Our response:** Thank you for the feedback. We have reviewed the reference list. There were some errors in the reference list (e.g. place of publication in reference# 1, 30, 33 and 34; semicolon in reference# 1, 6, 30, 33, 34; erratum in reference#3, 5 and 36; DOI in reference# 4, 8, 9, 20, 24, 27, 38, 41, 42 and 44; publisher name in reference# 40; book chapter format in reference# 11 and 39; author list in reference# 27 and journal name abbreviation in reference# 43) that have been corrected in the revised version of the manuscript. Sincere apologies for the unintentional errors. We confirm that we have not cited any retracted article in this manuscript.

**Reviewer: 1**

**Comment-1:**

In the methods section, line 96 to line 133 should be written in past tense. Read through the methods section to ensure its written in the correct tenses.

**Our response:** Thank you for the valuable comment. We have revised the methodology section to incorporate the suggestion. Please see lines 96-133.

**Additional changes:**

As part of this rebuttal, to ensure statistical integrity of the study findings, all statistical procedures were re-performed and reviewed. We subsequently identified that the method of computing dummy variable for age categories (i.e. Age groups 5-9 and 10-14) was incorrect and there was a calculation error in the ‘Number of associated impairments’ variable. As we have corrected these two variables, the unadjusted odds ratios for these variables (Table 3 [row 4 and 5] and Table 4 [row 30-32]) and adjusted odds ratios and corresponding *p* values for all variables entered into the multiple regression model (Table 5) have also changed to some extent. Considering the corrections, we have now revised the abstract (line 16-23), results (line 260, 267 and 271-277), discussions (line 289, 322-332 and 348-353) and conclusions (line 392) sections of the manuscript (unmarked version) accordingly. Additionally, the lower limit of 95% Confidence Interval of unadjusted odds ratios for Hearing Impairment has been changed from 1.7 to 1.6 (Table 4 [row 27]). We sincerely apologise for these unintentional errors that we have corrected now. However, we would like to confirm that the revised results have not made any major changes to our study findings.

Yours sincerely,

Gulam Khandaker

On behalf of the study investigators
